# Factors associated with diabetes concordant comorbidities among adult diabetic patients in Central Ethiopia: a cross-sectional study

**DOI:** 10.3389/fcdhc.2023.1307463

**Published:** 2023-12-13

**Authors:** Yohannes Mekuria Negussie, Mihiret Shawel Getahun, Nardos Tilahun Bekele

**Affiliations:** ^1^ Department of Medicine, Adama General Hospital and Medical College, Adama, Ethiopia; ^2^ Department of Nursing, Adama General Hospital and Medical College, Adama, Ethiopia; ^3^ Department of Public Health, Adama Hospital Medical College, Adama, Ethiopia

**Keywords:** concordant comorbidity, diabetes mellitus, diabetic epidemiology, adama, Ethiopia

## Abstract

**Background:**

Diabetes comorbidities are a serious public health issue that raises the risk of adverse health effects and complicates diabetes management. It also harms emotional health, medication adherence, self-management, and general quality of life. However, evidence is scarce in Ethiopia, particularly in the study area. Thus, this study aimed to estimate the prevalence of diabetes concordant comorbidities and identify factors associated with the presence of concordant comorbidities among adult diabetic patients in central Ethiopia.

**Methods:**

A health facility-based cross-sectional study was conducted among 398 adult diabetic patients. A computer-generated simple random sampling was used to select study participants. Data were collected using a structured data extraction checklist. The collected data were entered into Epi info version 7.2 and exported to SPSS version 27 for analysis. A binary logistic regression model was used to analyze the association between dependent and independent variables. An adjusted odds ratio with the corresponding 95% confidence interval was used to measure the strength of the association and statistical significance was declared at a p-value < 0.05.

**Result:**

The prevalence of diabetes-concordant comorbidities was 41% (95% CI: 36.2-46.0). The multivariable logistic regression model showed that age 41–60 (AOR = 2.86, 95% CI: 1.60–5.13), place of residence (AOR = 2.22, 95% CI: 1.33–3.70), having type two diabetes (AOR = 3.30, 95% CI: 1.21–8.99), and having positive proteinuria (AOR = 2.64, 95% CI: 1.47–4.76) were significantly associated with diabetes concordant comorbidities.

**Conclusion:**

The prevalence of diabetes-concordant comorbidities was relatively high. Age, place of residence, type of diabetes, and positive proteinuria were factors associated with diabetes-concordant comorbidities. Prevention, early identification, and proper management of diabetes comorbidities are crucial.

## 1 Introduction

Diabetes mellitus (DM) is a serious public health concern that is on the verge of epidemic proportions, making it a major global threat to the health and well-being of individuals, families, and society. About 537 million adults globally were anticipated to have DM in 2021, and this number is projected to rise to 643 million by 2030 and 783 million by 2045, with low- and middle-income countries holding the greatest burden of the forecasted increase in prevalence. In 2021, 24 million adults in Africa, or 1 in 22 adults, had DM. By 2045, it’s predicted to reach over 55 million (1–3).

With an estimated 1.9 million adults suffering from DM, Ethiopia is among the top five African nations in terms of the number of patients living with DM (3). Despite the lack of national statistics on diabetes prevalence, a systematic review and meta-analysis showed that it ranged from 2% to 6.5%. In Ethiopia, diabetes and its complications constitute the major causes of illness and mortality, with corresponding economic consequences (4).

Comorbidity refers to the presence of two or more chronic diseases or medical conditions in a patient (5). Diabetes comorbidity is the presence of one or more chronic diseases in patients living with DM, which can be divided into concordant and discordant comorbidities. Concordant comorbidities are two or more diseases that have a comparable pathophysiological risk profile and a similar disease treatment plan as DM. whereas, conditions that are not etiologically related to DM and don’t share comparable risk factors are discordant comorbidities (6–8).

Comorbidity harms a DM patient’s overall quality of life. Their emotional health, medication compliance, and self-management are all negatively impacted. It also increases the risk of adverse health outcomes and makes managing DM more demanding (9, 10). A recent study of more than 1.3 million participants found that over 98% had at least one comorbid condition, and nearly 90% had at least two (11). In sub-Saharan Africa (SSA), the prevalence of DM comorbidity ranges from 6% to 64% (12). A study done in Ethiopia also revealed that 55.8% of adults with DM had concordant comorbidities (6).

Previous studies have shown that various factors like sex, age, place of residence, economic status, duration of DM, family history of DM, type of DM, type of treatment, proteinuria, and glycemic control all influence the development of comorbidities in DM patients (6–8, 13–16).

Diabetes comorbidities increase the cost of hospital stays, the requirement for regular checkups, and the demand for healthcare. By being more cognizant of common diabetes comorbidities and related factors, it will be easier to decide on the appropriate course of treatment for DM patients. It has been demonstrated that early detection and effective comorbidity treatment can prolong the patient’s life and improve their quality of life (6, 12, 16, 17). However, there is a dearth of evidence in Ethiopia, particularly in the study area, to show the prevalence and factors associated with diabetes-concordant comorbidities. Hence, this study aimed to estimate the prevalence of diabetes concordant comorbidities and identify factors associated with the presence of concordant comorbidities among adult diabetic patients in central Ethiopia. The findings might be of essential clinical value, aiding in the comprehensive management of DM patients as well as designing policies and suitable intervention strategies. The findings of this study will serve as a basis for further studies as well.

## 2 Materials and methods

### 2.1 Study design, setting, and period

A health facility-based cross-sectional study was conducted at Adama Hospital Medical College (AHMC), Ethiopia. This study was conducted to retrieve five years of data, from January 2018 to December 2022. AHMC is the sole public teaching hospital in Adama town. It has more than 1300 staff, a yearly outpatient flow of 226,000 with an average outpatient flow of 853, 500 beds, and an admittance rate of 173 patients per week. Adama Town is 99 kilometers southeast of Addis Ababa, with a total area of 29.86 square kilometers and a population of over 500,000 people. The hospital collectively provides services to catchment areas with over five million people and acts as a referral hub for zones and regions nearby.

### 2.2 Population and eligibility criteria

All adults living with DM who were on follow-up at AHMC were considered the source population, whereas all adult diabetic patients who were on follow-up at AHMC from January 2018 to December 2022 were taken as the study population. Patients with an uncertain comorbidity status and/or an incomplete transfer-out history were excluded from this study.

### 2.3 Sample size determination and sampling procedure

The sample size for this study was determined by using the single population proportion formula by considering the assumptions of a 95% confidence level (the critical value Zα/2 = 1.96), a 5% margin of error (d = 0.05), and a 55.8% (p = 0.558) proportion of diabetes concordant comorbidity from a study done in Hiwot Fana Specialized University Hospital (6). By adding 10% contingency for incomplete data, the final sample size of the study becomes 421.

Using the medical record number of diabetes patients who were on follow-up from January 2018 to December 2022 as a sampling frame, participants who fulfilled the inclusion criteria were selected by a simple random sampling technique using computer-generated random numbers.

### 2.4 Study variables

#### 2.4.1 Dependent variable

Diabetes concordant comorbidities

#### 2.4.2 Independent variables

##### 2.4.2.1 Socio-demographic factors

Sex, age, place of residence, marital status, and occupational status.

##### 2.4.2.2 Clinical factors

Type of DM, type of treatment, proteinuria, glycemic control, body mass index (BMI), family history of DM, duration of DM, history of smoking, and follow-up miss.

### 2.5 Operational definitions

#### 2.5.1 Concordant comorbidity

the existence or absence of at least one chronic condition among individuals with diabetes. These include hypertension(HTN), obesity, dyslipidemia, chronic vascular disease (CVD), and/or chronic kidney disease (CKD) (6, 18, 19).

#### 2.5.2 Hypertension

a systolic blood pressure (SBP) of ≥ 140 mmHg or diastolic blood pressure (DBP) of ≥ 90mmHg (at least two recordings on separate days or four hours apart in a single day) or being treated for physician-diagnosed hypertension (20, 21).

#### 2.5.3 Obese

a body mass index (BMI) exceeding 30 kg/m^2^.

#### 2.5.4 Dyslipidemia

the presence of at least one of those conditions: high triglyceride levels (>150 mg/dl), high low-density lipoprotein cholesterol (LDL-C) levels (>130 mg/dl), low high-density lipoprotein cholesterol (HDL-C) levels (< 40 mg/dl in men or < 50 mg/dl in women), and high plasma total cholesterol levels (>200 mg/dl) or being treated for physician-diagnosed dyslipidemia (22).

#### 2.5.5 Chronic vascular disease

the presence of at least one of those conditions: an electrocardiogram (ECG) finding indicating ischemic heart disease, an echocardiography finding of ischemic heart disease, or being on treatment after being diagnosed by a physician with stroke or ischemic heart disease (23).

#### 2.5.6 Chronic kidney disease

the presence of at least one of those conditions: urine dipstick protein +1 at least twice in the past three months; serum creatinine level ≥ 1.2 mg/dl in males and ≥ 1.0 mg/dl in females at least twice in the last three months; or being on treatment after being diagnosed by a physician with CKD (24).

#### 2.5.7 Glycemic control

hemoglobin A1C (HgA1C) < 7%, or the last fasting blood sugar (FBS) level ≤ 130 mg/dl in the absence of documented HgA1C determined in less than three months, was regarded as good glycemic control; otherwise, it was considered poor glycemic control (19).

### 2.6 Data collection procedure and quality control

Data were collected using a structured and pre-tested checklist devised by reviewing patients’ charts, follow-up cards, DM registration books, electronic information databases, and previous similar studies. The checklist includes socio-demographic, clinical-related characteristics, and comorbidity histories. After two days of training on the data collection process, three trained nurses collected the data under the supervision of two public health officers.

Before actual data collection, the checklist was pre-tested on 5% of the projected sample size (n = 21) on records earlier than January 2018, and then adjustments and corrections were made accordingly. Moreover, the supervisors and principal investigator constantly monitored the completeness and consistency of the data during the data collection, and all gathered data were cross-checked during data entry to clarify any missing data.

### 2.7 Data processing and analysis

Following data coding and entry into Epi-Info Vision 7, the data were exported to Statistical Package for Social Sciences (SPSS) Version 27 for cleaning and analysis. Descriptive statistics were employed to describe the study population in light of relevant characteristics. The Shapiro-Wilk test was used to validate the normality assumptions for continuous variables. Binary logistic regression was used to model the association between the independent and the outcome variables. The statistical assumptions of the model i.e., independence of residuals, multi-collinearity, normality, linearity, and outliers, were examined, and no significant violations were found. A p-value of 0.25 was used as a cut-off value in the bivariable logistic regression model to select variables for multivariable logistic regression analysis to control the possible effects of confounders. The standard model-building approach was used to fit the model. The model’s fitness was assessed using Hosmer and Lemeshow’s goodness-of-fit test, and the result was not significant with a p-value of 0.16 (> 0.05), indicating that the model fits the data well. Using the variance inflation factor and tolerance, the multicollinearity between the explanatory variables was also examined, and it was found to be within a tolerable range. In the multivariable logistic regression, the adjusted odds ratio (AOR) with a 95% confidence interval (CI) was used to determine factors independently associated with diabetes concordant comorbidity. At this level, variables with a p-value less than 0.05 were considered statistically significant.

## 3 Results

### 3.1 Socio-demographic characteristics

A total of 398 DM patient records were reviewed and included in the final analysis, giving a response rate of 94.5%. The median age of the participants was 48 (IQR= 37-60), with a minimum age of 18 and a maximum of 85 years. One hundred fifty-one (54.02%) of the participants were male, 293 (73.62%) were urban residents and 334 (83.92%) were married (**Table 1**).

**Table 1:** Sociodemographic characteristics of diabetic patients at Adama Hospital Medical College, Central Ethiopia (n=398).

Variables	Category	Frequency	Percentage
**Sex**	Male	151	54.02
	Female	183	45.98
**Age**	≤ 40	140	35.18
	41-60	158	39.70
	> 60	100	25.12
	Median 48 (IQR= 37-60)		
**Place of residence**	Urban	293	73.62
	Rural	105	26.38
**Marital status**	Currently married	334	83.92
	Currently unmarried	64	16.08
**Occupational status**	Employed	163	40.95
	Merchant	94	23.62
	Farmer	56	14.07
	Retired	65	16.33
	Others *	20	5.03

* Housewife, Student, Daily laborer.

### 3.2 Clinical characteristics

Among the participants included in this study, 341 (85.68%) were type 2 DM patients, and 172 (43.22%) were taking oral hypoglycemic agents (OHAs). The majority of DM patients, 346 (86.9%), had poor glycemic control. In this study, the median duration of DM was 3.8 (IQR = 1.8–6.1), and of the participants, 280 (70.35%) had a history of missed follow-up (**Table 2**).

**Table 2:** Clinical characteristics of diabetic patients at Adama Hospital Medical College, Central Ethiopia (n=398).

Variables	Category	Frequency	Percentage
Type of DM	Type I	57	14.32
	Type II	341	85.68
Type of treatment	Oral hypoglycemic agents	172	43.22
	Insulin	96	24.12
	Both	130	32.66
Oral hypoglycemic agents	Metformin	139	46.03
	Glibenclimide	45	14.90
	Both	118	39.07
Protein urea	Positive	67	16.83
	Negative	331	83.17
Glycemic control	Good	52	13.10
	Poor	346	86.90
Body mass index (Kg/m^2^)	Underweight	43	10.81
	Normal (18.5-24.99)	157	39.45
	Overweight (25–29.99)	107	26.88
	Obese (≥30)	91	22.86
Family history of DM	Yes	27	6.78
	No	371	93.22
Duration of DM	< 5 years	254	63.82
	≥ 5 years	144	36.18
	Median 3.8 (IQR = 1.8-6.1)		
History of Smoking	Yes	10	2.51
	No	388	97.49
Follow-up miss	Yes	118	29.65
	No	280	70.35

DM, diabetes mellitus.

### 3.3 The prevalence of concordant comorbidity

The overall prevalence of diabetes concordant comorbidities was 41% (95% CI: 36.2-46.0). In a separate analysis, it was 14% (95% CI: 5.3-22.8) among type 1 and 45.5% (95% CI: 40.2-50.7) among type 2 DM patients. From this, 79 (48.5%) participants had HTN, 29 (17.8%) had obesity, 6 (3.7%) had dyslipidemia, 12 (7.4%) had CVD, 18 (11%) had CKD, and 19 (11.7%) had multi-comorbidity.

### 3.4 Factors associated with concordant comorbidities

In the bivariable analysis, age, place of residence, type of DM, type of treatment, proteinuria, and duration of DM showed significant association. After adjusting for potential confounders using multivariable binary logistic regression analysis age, place of residence, type of DM and proteinuria demonstrated a statistically significant association with concordant comorbidities.

Accordingly, compared to diabetic patients who are 40 years of age or younger, those patients in the age range of 41 to 60 had 2.86 times the odds of having concordant comorbidities (AOR = 2.86, 95% CI: (1.60-5.13). The odds of having concordant comorbidities were 2.22 times higher among urban residents compared to those who were rural residents (AOR = 2.22, 95% CI: 1.33-3.70). Type 2 DM patients had 3.3 times greater odds of having concordant comorbidities compared to type one diabetes patients (AOR = 3.30, 95% CI: 1.21-8.99). Moreover, DM patients with positive proteinuria had 2.64 times the odds of having concordant comorbidities than their counterparts (AOR = 2.64, 95% CI: 1.47-4.76) **(** **Table 3**).

**Table 3:** Factors associated with diabetes concordant comorbidities among diabetic patients at Adama Hospital Medical College, Central Ethiopia (n=398).

Variable	Category	Concordant comorbidity		COR (95% CI)	AOR (95% CI)
		No	Yes		
**Age (years)**	≤ 40	105 (75.00)	35 (25.00)	1	1
	41-60	73 (46.20)	85 (53.80)	3.49 (2.13-5.73) *	2.86 (1.60-5.13) **
	> 60	57 (57.00)	43 (43.00)	2.26 (1.31-3.93) *	1.76 (0.93-3.32)
**Residence**	Urban	160 (54.61)	133 (45.39)	2.08 (1.28-3.37) *	2.22 (1.33-3.70) **
	Rural	75 (71.43)	30 (28.57)	1	1
**Type of DM**	Type I	49 (85.96)	8 (14.04)	1	1
	Type II	186 (54.55)	155 (45.45)	5.1 (2.35-11.1) *	3.30 (1.21-8.99) **
**Type of treatment**	OHA	102 (59.30)	70 (40.70)	0.67 (0.42-1.05)	0.61 (0.37- 1.00)
	Insulin	69 (71.88)	27 (28.12)	0.38 (0.22-0.67) *	0.80 (0.39-1.65)
	Both	64 (49.23)	66 (50.77)	1	1
**Protein urea**	Positive	27 (40.30)	40 (59.70)	2.51 (1.47-4.29) *	2.64 (1.47-4.76) **
	Negative	208 (62.84)	123 (37.16)	1	1
**Duration of DM**	< 5 years	157 (61.81)	97 (38.19)	1	1
	≥ 5 years	78 (54.17)	66 (45.83)	1.37 (0.91-2.07) *	1.44 (0.91-2.30)

*Significant at p-value <0.25 in unadjusted logistic regression analysis, **significant at p<0.05 in adjusted logistic regression analysis, 1= Reference.

AOR, adjusted odds ratio; CI, confidence interval; COR, crude odds ratio; DM, diabetes mellitus; OHA, oral hypoglycemic agent.

## 4 Discussion

The present study sought to assess the prevalence of concordant comorbidities and identify factors associated with the presence of concordant comorbidities among adult diabetic patients in central Ethiopia. The prevalence of diabetes concordant comorbidities was relatively high. Age 41 to 60, being an urban resident, having type 2 DM, and having positive proteinuria were factors significantly associated with diabetes concordant comorbidities.

In this study, the overall prevalence of diabetes concordant comorbidities was 41% (95% CI: 36.2-46.0). This finding is in agreement with studies conducted in Germany(43%) (25) and Scotland (42.2%) (26). The prevalence of concordant comorbidities in this study is higher than studies done in the United States of America (7%) (27), Israel (16.6%) (7), and Australia (18.9%) (28). Conversely, the finding of the current study is lower than those of studies done in the United States of America (USA) (88.5%) (11), Spain (82%) (29), India (66%) (8) and Ethiopia (55.8%) (6). This disparity may be explained by differences in the study population; most earlier studies focused exclusively on type 1 or type 2 DM, whereas the current study included both types of DM (7, 8, 27, 29). Further, differences in sample sizes, socio-demographic, and study period disparities might be another possible explanation for the observed variations.

The odds of having concordant comorbidities were 2.86 times higher among DM patients in the age range of 41 to 60 compared with those who are 40 years of age or younger. this finding is supported by studies done in Ethiopia (6), Israel (7), Basque country (15) and Saudi Arabia (30). This can be explained by the fact Arteries tend to constrict and harden with age, resulting in a condition called arterial stiffening. This process can lead to blocked arteries, which can raise the risk of various complications, including hypertension and cardiovascular problems. People tend to have more sedentary lives as they get older, which causes difficulty managing cholesterol and increases the risk of dyslipidemia. Furthermore, age-related kidney alterations, such as a decrease in the number of filtering units (nephrons) and diminished kidney tissue, might also lead to hypertension and renal complications (31–33).

The finding of this study indicated that the odds of having concordant comorbidities were higher among urban residents compared to those who were rural residents. which is supported by the study done in China (34). This may be attributable to a sedentary lifestyle, unhealthy eating habits, less physical activity, and an increased rate of obesity that are common in urban settings, all of which raise the risk of concordant comorbidities (35, 36). Conversely, different studies have produced contradictory findings about the association between residence and concordant comorbidities (37). Uncertainty exists regarding the nature of the association between the place of residence and concordant comorbidities, and further studies are needed to draw that conclusion.

Type of DM was another significant factor associated with concordant comorbidities. Type 2 DM patients had 3.3 times greater odds of having concordant comorbidity compared to type 1 DM patients. This finding is similar to studies conducted in Ethiopia (6), Israel (7), and Italy (38). This might be explained by shared risk factors, insulin resistance, atherogenesis, and chronic inflammation, as well as the increased incidence of obesity and autonomic neuropathy among Type 2 DM patients (39, 40).

This study also showed a statistically significant association between proteinuria and DM concordant comorbidities. In line with studies conducted in Bangladesh (14), India (41), and Korea (42) DM patients with positive proteinuria had higher odds of having concordant comorbidity. This could be because proteinuria impairs the kidneys’ capacity to remove waste products. Consequently, proteins like albumin that should stay in the body seep into the urine, raising the risk of cardiovascular and renal problems. Salt and water retention due to damaged kidneys may raise blood volume and, as a result, blood pressure. Persistently high blood sugar levels also potentially damage the blood vessels in the retina, resulting in vision problems (43, 44).

## 5 Limitations of the study

Given the study’s cross-sectional design, determining whether independent and dependent variables are causally connected is difficult. There may be a potential selection bias in conducting this study in a third-tier reference center. Moreover, due to the retrospective nature of the study, it was not possible to investigate the impact of certain sociodemographic and behavioral factors, as well as perform an in-depth analysis of cardiovascular comorbidities.

## 6 Conclusions

The prevalence of diabetes-concordant comorbidities was relatively high. Age, place of residence, type of DM, and positive proteinuria were factors associated with diabetes-concordant comorbidity. Thus, patients with diabetes who have co-morbid conditions or are at risk of getting them, such as those who are older, have type 2 DM, or have positive proteinuria, should receive due attention. Furthermore, prospective studies are recommended.

## Data availability statement

The original contributions presented in the study are included in the article/supplementary material. Further inquiries can be directed to the corresponding author.

## Ethics statement

Ethical approval for the study was received from Adama Hospital Medical College's Institutional Review Board (IRB), and authorization to access data from medical records was secured from the hospital's medical director. The need for consent was waived by the IRB. Data confidentiality was maintained at all stages of study processes and was stored on a secure password-protected system. All the study's procedures were conducted in line with the principles of the Helsinki Declaration (45).

## Author contributions

YN: Conceptualization, Data curation, Formal Analysis, Funding acquisition, Investigation, Methodology, Project administration, Resources, Software, Supervision, Validation, Visualization, Writing – original draft, Writing – review & editing. MG: Writing – review & editing. NB: Supervision, Writing – review & editing.
